# Supplementary material for: Limited role of spatial self-structuring in emergent trade-offs during pathogen evolution
Source: Sci Rep. 2018 Aug 20;8:12476. doi: 10.1038/s41598-018-30945-1 (PMC6102235; doi:10.1038/s41598-018-30945-1)
Supplement: Supplementary file 1 — Supplementary Information [file 41598_2018_30945_MOESM1_ESM.pdf]

Supplementary Information for "Limited role of spatial self-structuring in emergent trade-offs during pathogen evolution"

Víctor Buendía[1,2], Miguel A. Muñoz[1], and Susanna Manrubia[3,4,*]

[1]Departamento de Electromagnetismo y Física de la Materia e Instituto Carlos I de Física Teórica y Computacional, Universidad de Granada, E-18071 Granada, Spain
[2]Dipartimento di Scienze Matematiche, Fisiche, e Informatiche, Università di Parma, via G.P. Usberti, 7/A - 43124, Parma, Italy
[3]Grupo Interdisciplinar de Sistemas Complejos (GISC), Madrid
[4]Programa de Biología de Sistemas, Centro Nacional de Biotecnología (CSIC), Madrid, Spain
[*]Corresponding author

April 11, 2018

Finite size effects

In our experiments with diffusion, we can observe the ordered and the disordered phases coexisting in a finite interval around τ_{I0}^c. This is due to the existence of stochastic fluctuations that arise for a finite system size L. We have checked that increasing the system size reduces the width of the interval, so in the thermodynamic limit $L \to +\infty$ we only have a point separating the two phases.

A different effect of finite systems regards the appearance of periodic patterns which we here describe. Their appearance is due to the use of periodic boundary conditions, which permit that the characteristic length of patterns couples with

system size L. In our experiments, we have taken L large enough so finite size effects can be neglected.

When we let the system evolve without diffusion, strains that emit infected waves with higher frequency are selected. As the frequency changes, it becomes more probable that a wave can "connect" two sides of the space, producing a linear front. Inside the linear front, strains tends to increase its infection time, leading to the formation of stripes. Eventually, the high R_0 achieved at some points in the stripe deforms it and breaks the wide fronts again. Some pictures of the process are shown in Figure 1.

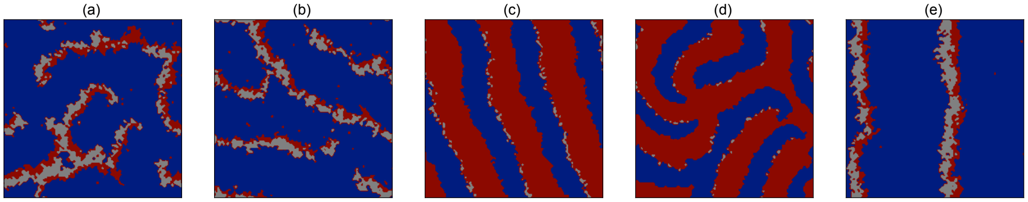

Figure 1: Finite size effects. We show the system configuration for large times, when finite size effects happen, for a system size $L = 100$. Time advances from left to right. (a) Spiral wave patterns before finite size effects start. (b) Spiral waves start to couple with system size, reaching both sides of space. (c) Wide linear fronts. (d) Collapse of the linear fronts into spirals. (e) Stable linear fronts.

To understand better this phenomenon, we have looked for a characteristic length of the system. This is done for several times t as follows:

1. Assign numerical values to the states, susceptible $\to 0$, infected $\to 1$, recovered $\to -1$ so we can operate with them. We have checked that the particular selection of values does not affect the results qualitatively.

2. Compute the two-dimensional Fourier transform of the system.

3. Compute the most important Fourier modes, $|k|$, and define a characteristic length as $\lambda = 1/|k|$.

If the evolutionary mechanism is not active, the characteristic length is always constant and depends on the emission frequency, meaning that λ can account for the evolution of the observed patterns. For the evolutionary trajectories, we have found that the characteristic length tends to increase with time, up to the point where the fronts form. After this point the characteristic length becomes constant.

We have also checked that the time needed to start producing the fronts increases as the system size N is increased. Therefore, our conclusion is that the front formation is a finite-size effect due to a coupling between a characteristic scale of the system and the system size. For this reason, in the experiments system size and timescales have been selected such that no finite-size effects can be seen. This effect was avoided in the original paper by Ballegooijen and Boerlijst [*Proc. Natl. Acad. Sci. USA* 101(52)18246–18250 (2004)] thanks to open boundary conditions. The nature of the boundary, therefore, does not play any role in the phenomenology discussed.
